# Evaluation of minimal fracture liaison service resource: costs and survival in secondary fracture prevention—a prospective one-year study in South-Finland

**DOI:** 10.1007/s40520-021-01826-x

**Published:** 2021-04-03

**Authors:** P. Lüthje, I. Nurmi-Lüthje, N. Tavast, A. Villikka, M. Kataja

**Affiliations:** 1 North-Kymi Hospital, Liljequistintie 13 B Kuusankoski, 45700 Kouvola, Finland; 2 Department of Public Health, University of Helsinki, Helsinki, Finland; 3 Kouvola Health Center, Kouvola, Finland; 4 National Institute for Health and Welfare, Helsinki, Finland

**Keywords:** One-year cohort, Low-energy fractures, Prospective study, Secondary prevention, Direct costs, Survival

## Abstract

**Background:**

Fracture liaison service (FLS) is a secondary prevention model for identification of patients at risk for fragility fractures.

**Aims:**

This study was conducted to evaluate the number and costs of secondary prevention of low-energy fractures in the city of Kouvola in Finland.

**Methods:**

Women aged ≥ 45 years and men ≥ 60 years treated in the emergency department with a low-energy fracture were identified. Laboratory testing, BMI, and DXA scans were performed. Fracture Risk Assessment Tool was used. The direct FLS costs were calculated. Survival was analyzed using univariate and multivariate analysis and the life-table method.

**Results:**

525 patients with 570 fractures were identified. The mean age of women was 73.8 years and of men 75.9 years. Most patients sustained wrist (31%), hip (21%) or proximal humerus (12%) fractures. 41.5% of the patients had osteoporosis according to DXA scans. 62% of patients used calcium and vitamin D daily and 38% started anti-osteoporotic medication. Protective factors for survival were: age < 80 years, female sex, and S-25OHD concentration of 50–119 nmol/L. Excess mortality was highest among patients with a fracture of the femur. The total annual direct costs of FLS were 1.3% of the costs of all fractures.

**Discussion:**

Many low-energy fracture types were associated with excess mortality. The use of anti-osteoporotic medication was not optimal.

**Conclusions:**

FLS increased the catchment of low-energy fracture patients and was inexpensive. However, identification, evaluation and post-fracture assessment of patients should be expedited. Rehabilitation of hip fracture patients needs to be improved.

**Supplementary Information:**

The online version contains supplementary material available at 10.1007/s40520-021-01826-x.

## Introduction

Osteoporosis is the most common disease of bone, characterized by reduced mineral density (BMD) and low bone strength, and leading to increased low-energy fracture risk. One-third of women and 20% of men over the age of 50 years will be affected by osteoporotic fracture in their lifetimes [1]. The prevalence of osteoporosis increases with age. Osteoporotic fractures are a remarkable public health problem due to the associated high morbidity and mortality [2].

The most common osteoporotic fracture is a wrist fracture. However, the most serious osteoporotic fracture is a hip fracture requiring surgical treatment. Hip fractures are associated with significant mortality at 30 days (7–8%) [3, 4] and at 1 year (14–39.5%) [5–8]. Patients with a hip fracture have a twofold risk of suffering a second hip fracture and their subsequent mortality is increased [9].

Vertebral fracture also is a major predictor of future fracture risk, up to fivefold for subsequent vertebral fracture and two- to threefold for fractures at other sites. It also leads to increased mortality [10].

There is a strong evidence indicating that early identification and treatment of osteoporosis are important factors in preventing subsequent fractures in the elderly [11, 12]. Secondary fracture prevention is important in improving the quality of life of aged people. Many studies have shown that fracture liaison services (FLS) has proved to be effective in reducing the frequency of subsequent fractures, in improving adherence to long-term anti-osteoporotic treatment, in decreasing morbidity of these patients and in providing cost savings [13–17].

Our aim was to explore the number of patients with low-energy fractures, to investigate secondary prevention among them, and to analyze the four-year survival of these patients, treated in North Kymi Hospital in the city of Kouvola, Finland in 2015. Moreover, we evaluated the direct costs of secondary prevention for the organization in charge. Thus far, no similar study has been published in Finland.

## Patients and methods

### Study design and population

We report the data and treatment of all low-energy fractures in women aged ≥ 45 years and men aged ≥ 60 years, according to the age limits of FLS programs at the time, treated in an in-patient unit or out-patient unit in North Kymi Hospital in 2015. Also, younger patients who had previous low-energy fractures before the index fracture were taken into account. The hospital is located in the city of Kouvola (86,000 inhabitants) in southeastern Finland (61°N).

Data were collected in the North Kymi Hospital Emergency Department (level II trauma center). The dedicated coordinating nurse identified the patients from the electronic patient records based on the hospital ICD-10 codes. Data included patients’ episodes based on the ICD-10 code, indicating typical osteoporotic fractures: proximal humerus, thoracolumbar spine, pelvis, hip, rib, and ankle as the primary diagnosis. Also, fractures of other locations in upper and lower extremities were included. The study period was from 1 January to 31 December 2015. Every 2 weeks the FLS-coordinator nurse received a list of all fracture patients (women aged ≥ 45 years and men ≥ 60 years), wherefrom she verified all low-energy fracture patients. Patients with high-energy fractures or pathological fractures were excluded.

### Fracture liaison service

Our FLS program was a nurse practitioner-led program. The FLS coordinator worked together with an orthopedic surgeon and traumatologist (PL). The screening service of the FLS coordinator nurse took about 70% of her full-time work, and the physician (PL) worked on a part-time basis. The other four FLS nurses worked on a part-time basis in local health centers and their patients were treated by local physicians. The coordinator nurse identified the patients and sent them a questionnaire on clinical risk factors.

Regarding in-patients who needed longer rehabilitation in health care, for example hip fracture patients or patients with a clinical vertebral fracture, the decision to prescribe anti-osteoporotic medication was made mainly by local physicians working in the rehabilitation department of the Kouvola Health Center, or in some cases by the orthopedic surgeon (PL). In all other cases the orthopedic surgeon (PL) prescribed anti-osteoporotic medication to the patients.

### Definition of variables

Patients were asked to give the following information: sex; age; date of injury; injury mechanism; and previous fractures. If they had sustained previous fractures, the type of fracture was inquired. Previous fractures were also obtained and confirmed from the electronic patient database established in 2004. Moreover, patients were asked questions about risk factors: parental history of hip fracture; familial history of osteoporosis; corticosteroid use; smoking; rheumatoid arthritis or connective tissue diseases; current use of medication including previous anti-osteoporotic medication; start time of anti-osteoporotic medication (date) and name of the medication; use of alcohol ≥ three drinks/day; previous DXA (dual-energy X-ray absorptiometry) examination and result; weight and height; and use of vitamin D or calcium or both supplements. According to Finnish guidelines [18], all patients gave samples that were laboratory tested to obtain the following information: sedimentation rate (SR); blood count; alkaline phosphatase (ALP); ionized calcium; creatinine; serum 25-hydroxyvitamin D (S-25OHD); transglutaminase antibodies; thyroid-stimulating hormone (TSH); and testosterone in men. Vitamin D deficiency was defined as a serum 25OHD concentration of < 50 nmol/L and optimal concentration for prevention of osteoporosis is 75–120 nmol/L [18].

### Fracture Risk Assessment Tool (FRAX)

Both the Finnish guidelines [18] and the Finnish practice [19] recommend that the 10-year fracture risk should be estimated using the proprietary Fracture Risk Assessment Tool to screen potential osteoporosis for testing bone mineral density. Additional risk factors such as frequent falls or frequent fractures, not represented in FRAX, require individual clinical judgment.

According to FRAX, fractures occurring at the spine, hip, distal forearm, and proximal humerus are considered major osteoporotic fractures (MOF). Therefore, the fractures were classified into two groups: (1) MOF and (2) other fractures.

An elevated 10-year probability score was defined as 3% or more for hip fracture and 20% or more for MOF.

### Bone mineral density (BMD)

BMD measurements were performed at the lumbar spine (L1–L4), total hip, and femoral neck by DXA using a Lunar densitometer (Prodigy, GE Medical Systems).

According to the WHO criteria, patients were classified based on the lowest T-score in the vertebrae L1–L4, total hip, and femoral neck. T-scores of ≤ − 2.5 standard deviations (SD) below the reference mean were classified as osteoporosis; T-scores between < − 1.0 and − 2.5 SD were classified as osteopenia; and T-scores ≥ − 1.0 SD were classified as normal.

According to Finnish national guidelines, hip fracture patients should be treated with anti-osteoporotic medication without BMD measurements if cancer or other reasons for secondary osteoporosis are excluded [18]. Anti-osteoporotic treatment is generally recommended for patients who have the bone mineral density T-score of -2.5 or less, or a history of spine or hip fracture. According to the Finnish Current Hip Fracture Care Guidelines, anti-osteoporotic treatment should only be considered if the patient would rehabilitate to independent mobility [20].

### Costs of FLS services

The immediate direct FLS costs for each patient were calculated using the cost of DXA scans in 2015; the cost of laboratory tests in 2015; the salaries for an FLS coordinator nurse working on a 70% basis in FLS; for other FLS nurses; and for a physician working on a part-time basis (about five hours every second or third week in 2015). Because the contributions of the other four FLS nurses were minor (30%), we used 100% of the annual salary for the FLS coordinator nurse. The DXA scan service was contracted from the private sector because North Kymi Hospital did not have a bone densitometer. The costs for all examinations of secondary fracture prevention were free of charge for the patient.

### Statistical analyses

Differences in the mean values between the groups were tested with t-test and two-way analysis of variance (ANOVA). For differences between two groups, the chi-squared (*χ*^2^) test, the Fisher’s exact test or the Wilcoxon rank-test were used. The *p* values < 0.05 were considered statistically significant.

### Survival

Cumulative mortality figures were counted by sex and fracture type at 1, 2, 3, and 4 post-fracture years. Four-year survival was analyzed using both the Bayesian multivariate model [21] and the life-table method [22]. In the univariate analysis, the odds ratios of survival were calculated for each class of variables and compared with each other within the variable. Statistical dependency within each variable was analyzed using the Chi-squared (*χ*^2^) test or the Wilcoxon rank test.

Multivariate analysis was performed using an optimizing step-wise procedure based on the Bayesian approach [21]. The optimizing procedure was developed mainly for categorized variables and it does not require a perfect variable matrix. The procedure selects, using a heuristic approach, the combination of variables that best explains the selected outcome variable. The Bayesian approach is applied by counting posterior probability ratios for each combination.

The aim was to find an optimal combination of variables that provides a better explanation than all the variables together. The relationship between the true positives and true negatives was graphically described as a receiver operating characteristic curve (ROC), for which the area under the curve (AUC) describes the approximate explanatory power of the model.

### Relative survival

Survival in relation to the reference population was analyzed according to sex and type of fracture using the life-table method [22]. In this method, the observed survival rates of the groups are compared with the survival rates based on sex- and age-specific life tables for the whole population of the same age and time period in Finland. The calculation of the survival of the reference population is 1.00. Thus, if the survival curve of the group remains below the survival of the reference population, there is an excess mortality in the group. Analysis of deaths was conducted based on information obtained from the nationwide administrative register, the Cause of Death Register of Statistics Finland.

The follow-up period started on 1 January 2015, and the closing date was 15 April 2020. The deaths were obtained from the Finnish Cause of Death Register. The follow-up time of survival was from 1 January 2015 to 15 April 2020 (63.5 months = 5.3 years). All patients were followed for a minimum of 51 months (4.3 years).

## Results

A total of 525 white native Finns (406 women and 119 men) with 570 low-energy fractures (see supplementary Fig. 1) were identified by the FLS nurses (70% by the FLS coordinator nurse and a total of 30% by four other FLS nurses). The mean age of women was 73.8 years (SD 12) and of men 75.9 years (SD 11) (n.s.) (Table 1). The median age in women was 50.5 years (38–98 years) and in men 54.1 years (43–97 years) (n.s.).

**Table 1:** Number of low-energy fractures among 525 patients and the mean ages according to previous fractures, side of index fractures and type of treatment

	Women (*n* = 406)		Men (*n* = 119)		Total (*n* = 525)		Statistic
	*n* (%)	Mean age (SD)	*n* (%)	Mean age (SD)	*n* (%)	Mean age (SD)	
Number and age of patients	406 (77.3)	73.8 (12.0)	119 (22.7)	75.9 (11.0)	525 (100)	74.3 (11.8)	n.s
Previous fractures (*n* = 418/525)							
Yes	157 (48.8)	75.6 (11.7)	33 (36.5)	75.3 (12.3)	192 (45.9)	75.5 (11.7)	
No	165 (51.2)	71.3 (11.7)	61 (63.5)	75.8 (10.8)	226 (54.1)	72.5 (11.6)	*F*-test 6.90, df 1;413,
Total	322 (100)	73.3 (11.9)	96 (100)	75.6 (11.3)	418 (100)	73.9 (11.8)	*p* < 0.01^b^
Site of index fracture (*n* = 525)							
Proximal humerus	50 (12.3)	75.0 (11.2)	15 (12.6)	74.7 (12.3)	65 (12.4)	74.9 (11.3)	
Other part of humerus	9 (2.2)	72.2 (7.8)	4 (3.4)	63.8 (1.9)	13 (2.5)	69.6 (7.6)	
Wrist	135 (33.3)	69.9 (11.0)	15 (12.6)	74.7 (7.2)	150 (28.6)	70.4 (10.8)	
Other part of forearm	17 (4.2)	70.6 (15.2)	3 (2.5)	64.9 (7.1)	20 (3.8)	69.7 (14.3)	
Vertebra	28 (6.9)	74.6 (11.2)	15 (12.6)	80.8 (11.3)	43 (8.2)	76.8 (11.5)	
Rib	11 (2.7)	82.2 (8.7)	7 (5.9)	67.0 (13.2)	18 (3.4)	76.3 (12.8)	
Pelvis	10 (2.5)	83.2 (5.6)	1 (0.8)	79.4	11 (2.1)	82.9 (5.4)	
Hip	72 (17.7)	81.6 (10.6)	41 (34.5)	79.7 (10.2)	113 (21.5)	80.9 (10.5)	
Other part of femur	9 (2.2)	83.4 (8.3)	4 (3.4)	78.8 (11.7)	13 (2.5)	82.0 (9.2)	
Tibia	19 (4.7)	70.5 (12.4)	4 (3.4)	76.2 (9.0)	23 (4.4)	71.5 (11.9)	*F*-test 9.72, df 11;510,
Ankle	44 (10.8)	67.5 (10.8)	9 (7.6)	69.8 (7.5)	53 (10.1)	67.9 (10.3)	*p* < 0.001^b^
Other fracture	2 (0.5)	82.7 (0.7)	1 (0.8)	59.7	3 (0.6)	75.0 (13.3)	*χ*^2^ = 31.02, df = 11,
Total	406 (100)	73.8 (12.0)	119 (100)	75.9 (11.0)	525 (100)	74.3 (11.8)	*p* < 0.01
Treatment (*n* = 548)^a^							
Conservative	249 (58.5)	73.2 (11.6)	65 (53.3)	74.6 (11.0)	314 (57.3)	73.5 (11.5)	
Operative	177 (41.5)	74.5 (12.6)	57 (46.7)	77.3 (10.8)	234 (42.7)	75.2 (12.3)	
Total	426 (100)	73.8 (12.0)	122 (100)	75.9 (11.0)	548 (100)	74.2 (11.8)	n.s
Inpatient treatment (*n* = 548)^a^							
Yes	218 (51.2)	76.8 (12.4)	76 (62.2)	78.5 (10.6)	294 (53.5)	77.2 (12.1)	
No	208 (48.8)	71.2 (11.0)	46 (37.8)	71.6 (9.8)	254 (46.5)	71.3 (10.8)	*F*-test 36.87, df 1;542,
Total	426 (100)	74.1 (12.1)	122 (100)	75.9 (10.9)	548 (100)	74.5 (11.9)	*p* < 0.001^b^

^a^23/525 patients had two or three accidents at different times, the total number of cases was 548 (426 women and 122 men)

^b^Vertical ANOVA

**Fig. 1:**
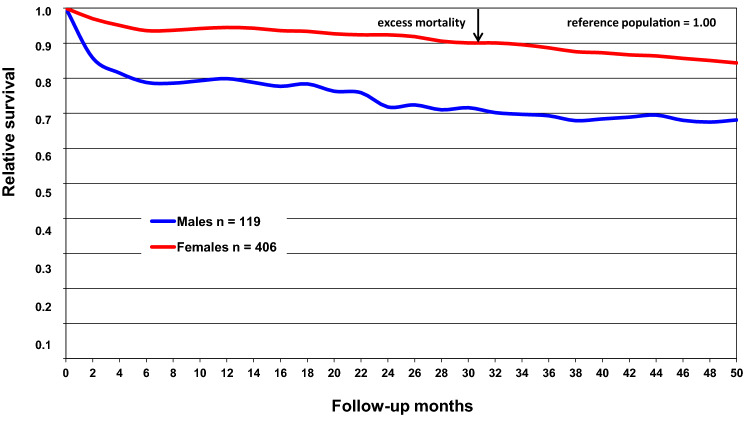
Survival of patients (*n* = 525) according to sex

Twenty-two patients (22/525) sustained two fractures in the same accident. In addition, 23/525 patients had two or three different falling accidents at different times. Therefore, the total number of cases was 548 (Fig. 1 and Table 1). These cases were mainly women (*n* = 20) and their mean age was higher than that of women with only one accident (80.4 years, SD 12.8 vs. 73.8 years, SD 12.0, respectively, *t* = 2.089, *p* < 0.05).

The distribution of the different fracture types according to sex is shown in Table 1. Women sustained more fractures than men. Every third woman and every seventh man sustained a wrist fracture (*χ*^2^ = 20.26, d.f. = 1, *p* < 0.001). One in three men and one in five women sustained a hip fracture (*χ*^2^ = 16.53, d.f. = 1, *p* < 0.001). The rate of MOF in women was 73.5% (313/426) and in men 73.0% (89/122).

Over half of the patients (54%) were treated as in-patient and 43% of the patients needed operative treatment. The mean age of patients by fracture type according to sex is shown in Table 1. In-patients were older than others, and patients with a fracture of the pelvis, hip or other part of the femur were older than those with other fractures (Table 1).

### Clinical risk factors

Previous fractures were the most common (46%) clinical risk factors reported by our patients in 2015 (Table 1), followed by current smoking (16.4%) (Table 2). All other clinical risk factors used in the FRAX algorithms were reported by 11.4% or less of these adults (Table 2). Patients with previous fractures were older than those without previous fractures (Table 1).

**Table 2:** Anti-osteoporotic medication prior to index fracture, clinical risk factors, vitamin D level, BMI, calcium plus vitamin D use and anti-osteoporotic medication after index fracture

	Women (%)	Men (%)	Total (%)	*p*
Anti-osteoporotic medication prior to index fracture (*n* = 374/525)^a^				
Yes	43 (14.4)	11 (14.5)	54 (14.4)	
No	255 (85.6)	65 (85.5)	320 (85.6)	n.s.
Total	298 (100)	76 (100)	374 (100)	
Parental history of hip fracture (*n* = 355/525)				
Yes	26 (9.2)	4 (5.6)	30 (8.5)	
No	258 (90.8)	67 (94.4)	325 (91.5)	n.s.
Total	284 (100)	71 (100)	355 (100)	
Smoking (*n* = 359/525)				
Yes	38 (13.2)	21 (29.2)	59 (16.4)	
No	249 (86.8)	51 (70.8)	300 (83.6)	*χ*^2^ = 10.63, d.f. 1,
Total	287 (100)	72 (100)	359 (100)	*p* < 0.01
Arthritis rheumatoides (*n* = 365/525)				
Yes	28 (9.6)	6 (8.2)	34 (9.3)	
No	264 (90.4)	67 (91.8)	331 (90.7)	n.s.
Total	292 (100)	73 (100)	365 (100)	
Corticosteroid use (*n* = 369/525)				
Yes	37 (12.5)	5 (6.8)	42 (11.4)	
No	258 (87.5)	69 (93.2)	327 (88.6)	n.s.
Total	295 (100)	74 (100)	369 (100)	
Alcohol use (≥ 3 drinks/day; *n* = 357/525)				
Yes	8 (2.8)	12 (16.9)	20 (5.6)	
No	278 (97.2)	59 (83.1)	337 (94.4)	*χ*^2^ = 21.40, d.f 1,
Total	286 (100)	71 (100)	357 (100)	*p* < 0.0001
Vitamin D level in nmol/L (*n* = 368/525)				
− 24	4 (1.4)	0 (0)	4 (1.1)	
25–49	19 (6.4)	6 (8.3)	25 (6.8)	
50–74	63 (21.3)	23 (31.9)	86 (23.5)	
75–120	150 (50.7)	28 (38.9)	178 (48.4)	
121–150	47 (15.9)	9 (12.5)	56 (15.0)	
151-	13 (4.4)	6 (8.3)	19 (5.2)	n.s.
Total	296 (100)	72 (100)	368 (100)	
BMI (kg/m^2^) (*n* = 324/525)				
< 18.5	7 (2.7)	3 (4.8)	10 (3.1)	
18.5–20.5	15 (5.7)	4 (6.5)	19 (5.9)	
20.6–25.0	92 (35.1)	22 (35.4)	114 (35.2)	
25.01–30.0	90 (34.4)	24 (38.7)	114 (35.2)	
30.01–35.0	44 (16.8)	5 (8.1)	49 (15.1)	
35.01-	14 (5.3)	4 (6.5)	18 (5.5)	n.s.
Total	262 (100)	62 (100)	324 (100)	
Calcium and vitamin D use after index fracture (*n* = 525)				
Yes	269 (66.3)	57 (47.9)	326 (62.1)	
No information	117 (28.8)	52 (43.7)	169 (32.2)	
No	20 (4.9)	10 (8.4)	30 (5.7)	*χ*^2^ = 13.27, d.f. 2,
Total	406 (100)	119 (100)	525 (100)	*p* < 0.01
Anti-osteoporotic treatment after index fracture (*n* = 386/525)				
Yes	120 (38.8)	26 (33.8))	146 (37.8)	
No	155 (50.2)	41 (53.2)	196 (50.8)	
Not accepted	18 (5.8)	6 (7.8)	24 (6.2)	
No information	16 (5.2)	4 (5.2)	20 (5.2)	n.s.
Total	309 (100)	77 (100)	386 (100)	
Anti-osteoporotic treatment among MOF patients who were alive 12 months after the index fracture				
Hip (women: n = 56; men: *n* = 22)	28 (50.0)	9 (40.9)	37/78 (47.4)	
Vertebra (women: *n* = 27; men: *n* = 11)	18 (66.7)	6 (54.5)	24/38 (63.2)	
Proximal humerus (women: *n* = 49; men: *n* = 13)	20 (40.8)	2 (15.4)	22/62 (35.5)	
Wrist (women: *n* = 131; men: *n* = 13)	29 (22.1)	5 (38.5)	34/144 (23.6)	n.s.
Total (women: 263; men = 59)	95 (36.1)	22 (37.3)	117/321 (36.4)	

*MOF* major osteoporotic fracture

^a^The questionnaire was returned by 374/525 patients. The differences in figures in Table 2 were due to missing information in the questionnaires. In some cases missing information was found in patients’ medical records

Among hip fracture patients, 35% (40/113) had sustained a total of 74 previous fractures. Three out of 113 hip fracture patients sustained a second hip fracture of the contralateral hip.

Of the patients who returned the questionnaire, and according to the electronic medical records, 46% (192/418) had sustained a total of 287 previous fractures: 123 patients had one, 51 patients had two, 12 patients had three, four patients had four, and two patients had sustained five previous fractures.

### Abnormal laboratory results

There were 20 patients (3.8%, 20/525) with abnormal laboratory results who were referred to an internist. In eight cases the reason was elevated calcium and parathormone (PTH) result, in four cases elevated ALP result, in two cases elevated anti-transglutaminase antibodies, and in six cases the reason was many previous fractures. In two cases the patient had a celiac disease, in one case primary sclerosing cholangitis and in one case parathyroid adenoma which was treated operatively. The rest 16 patients remained in the follow-up of the internist. None of the male patients had too low testosterone levels, which would require testosterone treatment. There was no secondary osteoporosis among the six patients with several previous fractures.

### Vitamin D, BMI and BMD

The S-25OHD concentration was measured in 368 patients (70%). In half of these patients (178/368, 48%), the S-25OHD level was optimal (75–120 nmol/L) (Table 2). The mean S-25OHD concentration was 93.4 nmol/L (SD 36.1). Among 113 hip fracture patients, the mean S-25OHD concentration was 94.5 nmol/L (SD 34.7).

The body mass index (BMI) (kg/m^2^) could be calculated in 324 patients (62%). BMI was normal in 41% (18.5–25.0 kg/m^2^) of these patients (Table 2). The mean value was 26.3 kg/m^2^, almost equal in both sexes. The mean BMI value was lowest among hip fracture patients and highest among vertebral fracture patients (*p* < 0.01).

The questionnaire was returned by 374 patients (71%), of whom in 66% (246/374) had their BMD measured. In 34% (128/374), the measure was not necessary due to fracture type, scarce risk-scores, or age. Of all the BMD-measured patients, 41.5% (102/246) (82 women and 20 men) had osteoporosis, and 52.8% (130/246) (112 women and 18 men) had osteopenia. In 14 patients (5.7%), the BMD result was normal.

### FRAX

The FRAX tool was in use in 62% (323/525) of cases. Approximately 67% of women (176/261) and 63% of men (39/62) had a hip fracture probability that was 3% or more. About 27% of the patients [87/323; 33% in women (86/261); 1/62 (1.6%) in men] had a MOF probability that was 20% or more.

### Use of calcium plus vitamin D

Of all patients, 62% used calcium plus vitamin D supplements (Table 2) at a minimum dose of 800 IU vitamin D per day. Calcium plus vitamin D was used by 47% of hip fracture patients, 76% of vertebral fracture patients, 68% of wrist fracture patients and 66% of proximal humerus fracture patients. Daily calcium supplementation was recommended if the patient was unable to achieve an intake of 1200 mg/day of calcium from food sources.

### Anti-osteoporotic treatment

According to the questionnaire, 14% of patients used anti-osteoporotic medication prior to the index fracture, mostly bisphosphonates (86%) (Table 2). Post-fracture anti-osteoporotic medication was started on average 200 days after the index fracture in 38% of the patients (Table 2). Before starting the anti-osteoporotic treatment (bisphosphonates or denosumab), patients were recommended for dental examination for possible periodontal diseases. An antiresorptive medication was started after the dentist’s approval.

The total use of anti-osteoporotic medication, and among MOF patients who were alive 12 months after the index fracture, is shown in Table 2. Overall, 36% of the MOF patients still used anti-osteoporotic medication at 1 year after the fracture.

In most cases, subcutaneous denosumab (60 mg every 6 months) (47.5%) was used followed by oral bisphosphonates (38%) (alendronate 70 mg or risedronate 35 mg once a week), intravenous zoledronic acid 5 mg (13.5%), oral strontium ranelate 2 g/day (1%), and subcutaneous teriparatide 20 µg/day (1%).

The primary drug option for patients with hip fracture was intravenous zoledronic acid (5 mg per year, three times) to avoid compliance problems, and it was free of charge for the patient. Half of the hip fracture patients started with intravenous zoledronic acid, 41% with subcutaneous denosumab, and 8% with oral bisphosphonates.

### Costs in 2015

The annual salary of the FLS coordinator nurse was 32 900 € and the salary of the physician (part-time basis) was 5 100 € (70 €/h). The costs of laboratory tests were 52.23 €/patient for women and 73.75 €/patient for men. The cost for men was higher than for women due to testing testosterone. The total laboratory costs for 294 women and in 72 men totaled 20 666 €. The cost for one BMD measurement (lumbar spine and hip) was 110 €. Thus, the total costs for all DXA scans (*n* = 246) totaled 27 060 €. Altogether, the cost for FLS totaled 85 726 € in the year 2015.

### Mortality

The mortality of both sexes with different fracture types was studied separately. The highest one-year mortality among males was in those with hip fractures (46%) and vertebral fractures (27%). In females, the highest one-year mortality was among those with femoral shaft or distal femur fracture (44%) and pelvis fractures (30%) (Table 3). The four-month and one-year mortality among hip fracture patients were 25% and 31%, respectively.

**Table 3:** Cumulative mortality (at 1, 2, 3 and 4 years) among 525 patients with low energy fractures according to sex and fracture type

	Males (*n* = 119)^a^								Females (*n* = 406)^a^								All (*n* = 525)^a^	
	1 year		2 year		3 year		4 year		1 year		2 year		3 year		4 year		4 year	
	*n*	%	*n*	%	*n*	%	*n*	%	*n*	%	*n*	%	*n*	%	*n*	%	*n*	%
Proximal humerus	2	13	5	33	7	47	8	53	1	2	4	8	7	14	11	22	19	29
Other part of humerus	0	0	1	25	2	50	2	50	1	11	1	11	2	22	2	22	4	31
Wrist	2	13	3	20	3	20	4	27	4	3	8	6	13	7	18	13	22	15
Other part of forearm	0	0	1	33	1	33	1	33	0	0	2	12	2	12	4	24	5	25
Costa	0	0	1	14	1	14	1	14	2	18	2	18	3	27	6	54	7	39
Vertebra	4	27	6	40	7	47	7	47	1	4	4	14	5	18	7	25	14	33
Pelvis	0	0	0	0	0	0	0	0	3	30	4	40	4	40	5	50	5	45
Hip	19	46	23	56	25	61	28	68	16	22	23	32	34	47	39	54	67	59
Other part of femur	1	25	1	25	1	25	1	25	4	44	5	56	6	67	7	78	8	62
Tibia	1	25	1	25	1	25	1	25	3	16	3	16	4	21	4	21	5	22
Ankle	0	0	0	0	0	0	0	0	1	2	2	5	3	7	4	9	4	8
Other fracture	0	0	0	0	0	0	0	0	0	0	0	0	0	0	0	0	0	0
Total	29	24	42	35	48	40	53	44	36	9	58	14	83	20	107	26	160	30

^a^Total numbers of the 12 fracture types according to sex are presented in Table 1

At the end of the follow-up (4 years), the highest mortality among males was noticed in those with hip fractures (68%) and proximal humerus fractures (53%). Correspondingly, the highest mortality among females was found in those with femoral shaft or distal femur fractures (78%) and hip fractures (54%) (Table 3).

In total, 70% (365/525) of the patients survived 4 years after the index fracture (Table 3). Differences in mortality between sexes were found at 4 years. Mortality was higher among men than women in proximal humerus fractures (Fishers exact *p* = 0.0435) and in hip fractures (Fisher's exact *p* = 0.0001).

### Survival

Univariate analysis included the following variables: sex, age, S-25OHD concentration level, and type of fracture (Table 4).

**Table 4:** Univariate analysis of four variables^a^ in relation to survival data (*n* = 525)

Variable	Negative		Positive	Total	OR	95% CI	Statistic
Sex							
Male	62		57	119	2.19	1.45–3.31	
Female	286		120	406	0.46	0.30–0.69	*χ*^2^ = 13.86, *df* = 1, *p* < 0.001
Age							
< 60	61		0	61	0.01	0.003–0.06	
60–69	112		19	131	0.25	0.16–0.71	
70–79	117		36	153	0.50	0.28–0.85	
80–89	52		84	136	5.14	3.08–9.03	
≥ 90	6		38	44	15.58	7.75–31.32	Wx = 11.81, *p* < 0.001
S-25OHD concentration level^b^							
− 49		22	7	29	1.12	0.46–2.71	
50–74		72	14	86	0.61	0.33–1.15	
75–119		138	35	173	0.80	0.49–1.31	
≥ 120		54	26	80	2.00	1.16–3.44	Wx = 1.886, n.s
Type of fracture (*n* = 522)^c^							
Pelvis		6	5	11	1.66	0.50–5.44	
Humerus		52	26	78	0.98	0.61–1.57	
Wrist		122	28	150	0.35	0.22–0.55	
Vertebra		28	15	43	1.06	0.56–2.02	
Hip		43	70	113	4.64	3.05–7.06	
Ankle		48	5	53	0.18	0.08–0.43	
Costa		10	8	18	1.60	0.63–4.10	
Forearm		14	6	20	0.84	0.32–2.21	
Shaft or distal femur		5	8	13	3.25	1.12–9.52	
Tibia		18	5	23	0.53	0.20–1.44	*χ*^2^ = 70.68, *df* = 9, *p* < 0.001

*χ*^2^ Chi-squared test, *df* degrees of freedom, *OR* odds ratio, *n.s.* not significant, *S-25OHD* serum hydroxyvitamin D, *Wx* Wilcoxon rank test

^a^All the classes of the variables are compared with each other within the variable

^b^*n* = 368

^c^Three small bone fractures were omitted

In the multivariate analysis (Table 5), the most protective factors were: age under 80 years, female sex, and S-25OHD concentration of 50–119 nmol/L. The model correctly predicted 80.2% of the cases. The sensitivity and specificity of the rule were 79.1% and 80.7%, respectively (see supplementary Table 1). The *κ* value was 0.58 (95% CI 0.50–0.65), i.e. moderate. The graphic description of the model (AUC) is shown in supplementary Fig. 2.

**Table 5:** The most important factors (in italics) explaining survival selected out of three variables in 525 patients

RR-limit	False negative count	Sensitivity	Specificity	κ	Added variable
0.51	55	68.9	83.3	0.520	*Age (< 80 years)*
0.56	37	79.1	77.9	0.540	*Sex (female)*
0.88	37	79.1	80.7	0.575	*S-25OHD concentration (50–119 nmol/L)*
	36	79.7	77.3	0.538	Type of fracture

**Fig. 2:**
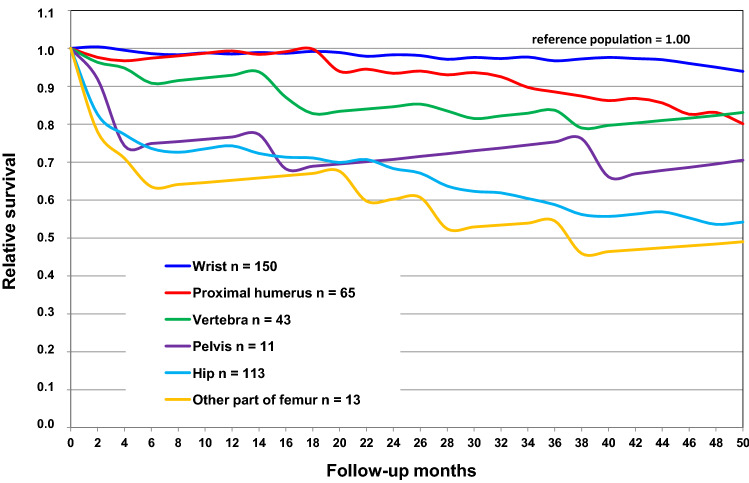
Survival of patients (*n* = 525) according to fracture type

In the total data, survival in relation to the reference population was higher among women than men (Fig. 1). There was no significant difference either in the mean age or in the median age between males and females. Survival varied according to the fracture type (Fig. 2). Patients with a fracture of the femoral shaft or distal femur had the lowest rate of survival followed by patients with a hip fracture, pelvis fracture and vertebral fracture, respectively. There was no excess mortality among patients with forearm, wrist or ankle fracture.

## Discussion

Our FLS program indicates the necessity of this service: about 75% of the patients belonged to the MOF category, nearly half (46%) of the patients had previous fractures, and more than one out of three (36%) of them had two to five previous fractures. Moreover, among hip fracture patients the rate of previous fractures was high (35%). Nevertheless, according to the questionnaire, only 14% of our patients used anti-osteoporotic treatment before the index fracture.

The treatment of patients with osteoporotic fractures is expensive. According to a US study, in women aged 55 years and older, the hospitalization burden of these fractures and population facility-related hospital costs were higher than those of either myocardial infarction, stroke, or breast cancer [23]. In our study, the one-year treatment costs of 116 hip fracture patients for the city of Kouvola (86 000 inhabitants) totaled 3.52 million euros in the study year 2015 (€ 30 338/patient) based on the Finnish PERFECT Hip Fracture Database [24]. According to a study in the European Union, hip fractures were estimated to account for 54%, and other osteoporotic fractures for 46% of the costs of all osteoporotic fractures in the EU [25]. Based on that estimation, the total costs of all osteoporotic fractures totaled about 6.52 million euros in our study in 2015 for the city of Kouvola. However, the direct costs for secondary fracture prevention were only 1.3% (€ 85 726) of the total annual costs.

Subsequent fractures can be prevented if osteoporotic fractures are identified and treated. A recent Swedish national register study showed that in women aged ≥ 50 years with a fragility fracture at any skeletal location, the incidence of subsequent fractures within 12 months was 7.1%, increasing to 12% at 24 months [26]. A similar result was found in postmenopausal women in the Netherlands: the absolute risk of any subsequent fracture in the year following the first fracture was 6% [27]. In a large US study involving women, the incidence of any subsequent fracture was 10% within 1 year of initial fracture [28].

In the present study, 47% of all hip fracture patients who survived 12 months after the index fracture used anti-osteoporotic medication. According to the Finnish Current Hip Fracture Care Guidelines, anti-osteoporotic treatment should only be considered if the patient would rehabilitate to independent mobility [20]. A recent Finnish hip fracture study among 538 home-dwelling patients showed that 9% of the patients were in permanent institutional care 12 months after the index fracture [29].

Moreover, in our present data the use of anti-osteoporotic treatment among patients who were alive 12 months after the index fracture was 63% in those with clinical vertebral fracture, 36% in those with proximal humerus fracture, and 24% in those with wrist fracture. Of all patients, 62% used calcium plus vitamin D supplements daily (at least 800 IU vitamin D per day).

Post-fracture use of anti-osteoporotic medication in our data was not optimal. The main reason was a long-lasting public discussion on overdiagnosing bone fragility and overtreating osteoporosis in various Finnish media sources during the study year 2015. These arguments have also been published in 2015 [30]. All this had a potential to discourage patients with low-energy fractures undergoing fracture preventive measures and anti-osteoporotic treatment. Moreover, many patients in Finland quit their anti-osteoporotic medication.

A recent study from Denmark, Catalonia and the UK in 2005–2015 showed a remarkable treatment gap in anti-osteoporotic medication among elderly patients with a first osteoporotic index-fracture: in Denmark 88–90%, in Catalonia 80–88% and in the UK 63–73% were not treated with anti-osteoporotic medication [31].

In our previous study (2003–2004), the mean S-25OHD concentration was 38.1 nmol/L among female and 37.0 nmol/L among male hip fracture patients [32]. In the present study, the mean concentration in both sexes was 2.5 times higher than 11 years ago, and among 113 hip fracture patients the mean S-25OHD concentration was 94.5 nmol/L (SD 34.7). This indicates that vitamin D deficiency among the elderly is not as common as it was in Finland 11 years ago. This result is supported by a recent prospective study using national data on Finnish adults aged ≥ 30 years with an 11-year follow-up [33]. The mean S-25OHD level among those aged ≥ 75 years-old increased from 43 nmol/L in 2000 to 65 nmol/L in 2011, and the prevalence of vitamin D deficiency decreased from 65.5 to 9.7% during the same time period [33].

Our study showed a higher long-term survival rate among patients with a sufficient (50–74 nmol/L) and optimal S-25OHD level (75–119 nmol/L). Many studies have shown that a low concentration of S-25OHD is associated with increased risk of death from all causes. A European meta-analysis of eight prospective studies with 26 916 study participants (median age 61.6 years) and a median S-25OHD concentration of 53.8 nmol/L, showed that participants with a concentration of 75–99 nmol/L had the lowest all-cause mortality. Low S-25OHD level was significantly associated with all-cause mortality and cardiovascular mortality [34]. The median follow-up time was 10.5 years [34].

A nationwide register-based study from Denmark reported that hip fractures were associated with the highest excess mortality (33% in men and 20% in women at 1 year after fracture). One-year excess mortality after fracture of the femur or pelvis was 20–25%; after vertebral fractures 10%; after humerus, rib, or clavicle fractures 5–10%; and after lower leg fractures 3% [35]. In the present study, the highest excess mortality at 1 year was among female patients with femoral shaft or distal femur fracture (44%), and among male hip fracture patients (44%), followed by male vertebral fracture patients (26%), and by female hip fracture patients (22%).

The mortality of hip fracture patients in the present study was high at 4 months and 1 year: 25% and 31%, respectively. Patients’ standard geriatric rehabilitation was not optimal. In the study-year (2015), 114 hip fracture patients (not included in the present data) living in South-Kymenlaakso were operated on the central hospital in the city of Kotka. They received post-operative multi-disciplinary rehabilitation at the Hoiku Rehabilitation Center. Their mortality at 4 months and 1 year was 15% and 19%, respectively. A recent meta-analysis on one-year mortality rate post hip fracture suggested 22% to be an expected mortality rate [36].

Some results of the present study were published in Finnish language in November 2017 [37]. Quite soon after publication, patients’ waiting time to dental care in the study region remarkably shortened enabling to start anti-osteoporotic medication as soon as possible. It is important to avoid any delay in starting the medication to decrease the risk of subsequent fractures [38]. Furthermore, since November 2019 all hip fracture patients in the city of Kouvola have received post-operative multidisciplinary rehabilitation at the above-mentioned Hoiku Rehabilitation Center. A systematic review and meta-analysis showed that in comparison to usual care, general or orthopedic geriatric rehabilitation programs improved patients’ functional status and reduced their admissions to nursing homes and mortality [39].

### Strengths and limitations

The strengths of our study were the long follow-up time, detailed patient-specific data, and the large number of consecutive patients at a single institution. We received a remarkably high response rate to the questionnaire (71%, 374/525). Usually, lower response rates are reported for pencil and paper studies [40]. The high response rate showed that our FLS had succeeded quite well.

We are convinced that most of the low-energy injuries in the study area have been treated in the study hospital because it is the only acute care hospital in this region and all X-rays ordered by the primary health centers are performed at this hospital. Some of the injury cases may have been treated at local private clinics and are not included in these data. We believe that these cases are rare, because in Finland elderly injured patients are usually treated in the emergency departments of acute care hospitals.

Only native-born Finns were included in our data and no other ethnic groups were represented. The foreign-born population in Finland is small (6%) and less than 3% of foreign-born persons lived in the study area in 2015 (Statistics Finland). The ethnicity, race, and different cultural background of foreign-born individuals may influence the prevalence of low-energy fractures. In Sweden [41] and Norway [42], for example, there was a reduced risk of hip fracture in foreign-born individuals.

Our study has several limitations. It is a single-center study, and therefore, the results cannot be generalized to a larger population. There was no control group without fracture liaison services, either. Moreover, the long-term use of anti-osteoporotic treatment was not evaluated. Gathering data from other centers would make the results more reliable. However, our study city was the 10th largest city in Finland (a country with 5.5 million inhabitants) in 2015.

## Conclusions

About 75% of all patients belonged to MOF category. Previous fractures were sustained by nearly half of all patients, of whom more than one in three patients had two to five previous fractures. Our minimal FLS resource increased the catchment of low-energy fracture patients and the FLS also was inexpensive. The total direct costs for FLS in the study year were only 1.3% of the annual total costs of all low-energy fractures in the study area. Hip fracture patients need multidisciplinary rehabilitation for optimal recovery to minimize the post-operative mortality during the first year after the index fracture. In total, anti-osteoporotic medication was started in 38% of patients. This was not optimal considering the main goal of FLS, which is to reduce fracture risk and subsequent fractures. Differences in mortality between sexes were found at 4 years. Mortality was significantly higher among men than women in proximal humerus fractures and in hip fractures. The excess mortality during the first four-year post-fracture follow-up time was highest among patients with femoral fractures.

## Supplementary Information

Below is the link to the electronic supplementary material.

Supplementary Fig. 2 Receiving Operating Characteristic (ROC) curve of the multivariate model area under curve (AUC): 0.840 (PDF 11 KB)

Supplementary Table 1 (DOCX 12 KB)

Supplementary Fig. 1 Flowchart depicting patient and fracture collection (PDF 10 KB)
