# Supplementary figures and images for: Evaluation of minimal fracture liaison service resource: costs and survival in secondary fracture prevention—a prospective one-year study in South-Finland
Source: Aging Clin Exp Res. 2021 Apr 3;33(11):3015–27. doi: 10.1007/s40520-021-01826-x (PMC8595226; doi:10.1007/s40520-021-01826-x)

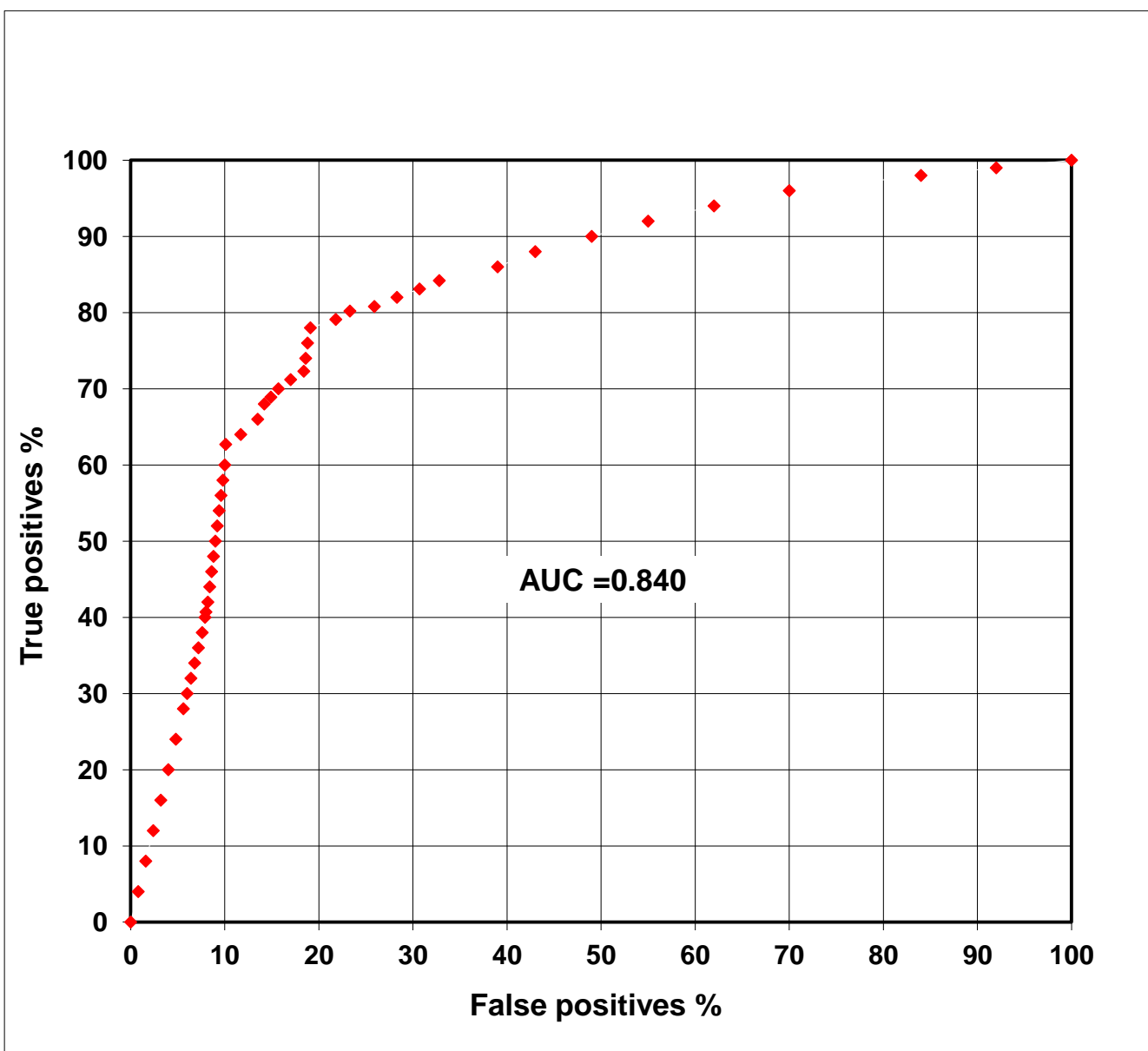

Supplement: Supplementary file 1 — Supplementary Fig. 2 Receiving Operating Characteristic (ROC) curve of the multivariate model area under curve (AUC): 0.840 (PDF 11 KB) [file 40520_2021_1826_MOESM1_ESM.pdf]

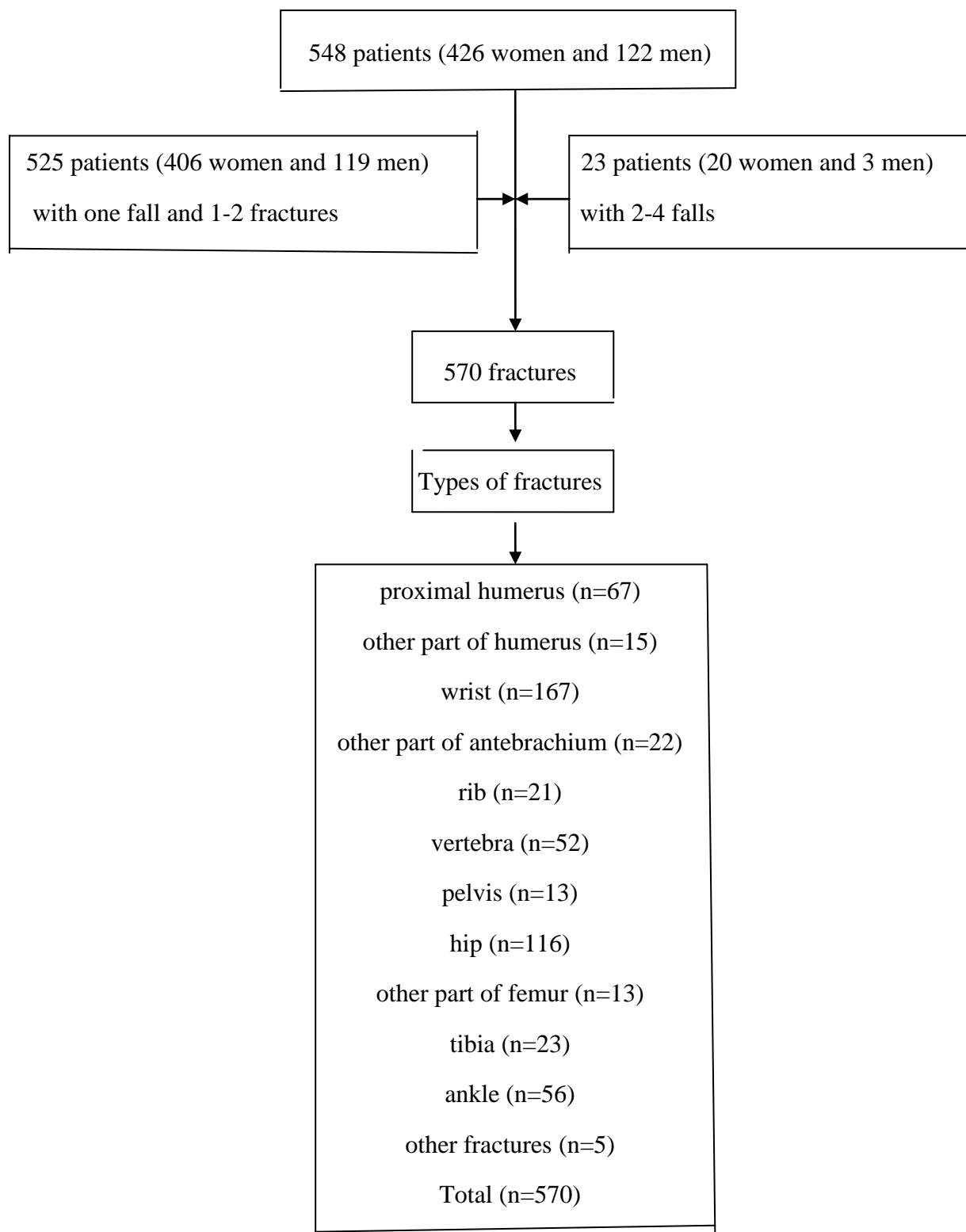

Supplement: Supplementary file 3 — Supplementary Fig. 1 Flowchart depicting patient and fracture collection (PDF 10 KB) [file 40520_2021_1826_MOESM3_ESM.pdf]
