# Supplementary material for: Evaluation of minimal fracture liaison service resource: costs and survival in secondary fracture prevention—a prospective one-year study in South-Finland
Source: Aging Clin Exp Res. 2021 Apr 3;33(11):3015–27. doi: 10.1007/s40520-021-01826-x (PMC8595226; doi:10.1007/s40520-021-01826-x)
Supplement: Supplementary file 2 — Supplementary Table 1 (DOCX 12 KB) [file 40520_2021_1826_MOESM2_ESM.docx]

Supplementary Table 1. Optimum result after three variables used

| Observed | Predicted | |  |
| --- | --- | --- | --- |
|  | Negative | Positive | Total |
| Negative | 281 | 67 | 348 |
| Positive | 37 | 140 | 177 |
| Total | 318 | 207 | 525 |
| κ= 0.58 [95% CI 0.50-0.65] (moderate); AUC (area under curve) 0.84 (Supplementary Figure 2)  Critical score=0.99; corresponding probability=49.6 %; sensitivity of the rule 79.1%; specificity of the rule 80.7 %; predicted value for negative prognosis 88.4 %; predicted value for positive prognosis 67.6 %; efficiency (correct prediction) 80.2 %; total error rate 19.8 % | | | |
